# The polymerase δ-interacting protein family and their emerging roles in diseases

**DOI:** 10.3389/fmed.2022.1026931

**Published:** 2022-11-08

**Authors:** Peiluo Huang, Lei Wu, Ningxia Zhu, Hongtao Zhao, Juan Du

**Affiliations:** ^1^Department of Immunology, College of Basic Medicine, Guilin Medical University, Guilin, China; ^2^College of Pharmacy, Guilin Medical University, Guilin, China; ^3^College of Continuing Education, Guilin Medical University, Guilin, China; ^4^Department of Pathophysiology, College of Basic Medicine, Guilin Medical University, Guilin, China

**Keywords:** DNA polymerase δ, POLDIP1, POLDIP2, POLDIP3, signaling pathway

## Abstract

The polymerase δ-interacting protein (POLDIP) family is a new family that can interact with DNA polymerase δ (delta). The members of the POLDIP family include POLDIP1, POLDIP2, and POLDIP3. Screened by the two-hybrid method, POLDIP1, POLDIP2, and POLDIP3 were initially discovered and named for their ability to bind to the p50 subunit of DNA polymerase δ. Recent studies have confirmed that POLDIPs are involved in the regulation of signal transduction pathways in neurodevelopment, neuropsychiatric diseases, cardiovascular diseases, tumors, and other diseases. However, each protein participates in different signaling pathways. In this review, we elucidate upon the family in terms of their genes and protein structures, their biological functions, in addition to the pathways that they are involved in during the development of diverse diseases. Finally, to provide new insights to the scientific community, we used the TCGA database to analyze and summarize the gene expressions of POLDIP family members in various tumors, as well as the correlations between their expressions and the overall survival times of tumor patients. Our data summary will give researchers working on cancer new concepts.

## Introduction

Polymerase δ-interacting protein (POLDIP) is a new family that interacts with DNA polymerase (delta). POLDIP1, POLDIP2, and POLDIP3 are members of the POLDIP family. POLDIP1, POLDIP2, and POLDIP3 were initially discovered and named for their ability to bind to the p50 subunit of DNA polymerase using the two-hybrid method (1, 2). Recent research has confirmed that POLDIPs play a role in the regulation of signal transduction pathways in neurodevelopment, neuropsychiatric diseases, cardiovascular diseases, tumors, and other diseases. However, each protein participates in a different signaling pathway. In this review, we elucidate the family in terms of their genes and protein structures, biological functions, and pathways that they are involved in during the development of various diseases.

**FIGURE 1:**
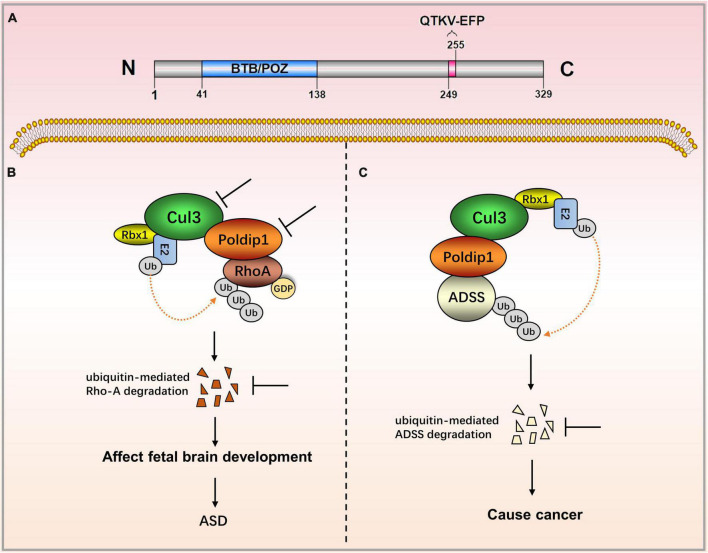
The role of POLDIP1 in the modulation of disease-related signaling pathways. **(A)** Domain map of POLDIP1. Its N-terminal has a BTB/POZ domain (residues 41–138) that is involved in the ubiquitination and degradation of RhoA. The C-terminal possesses the motif QTKV-EFP (residues 249–255), a proliferating cell nuclear antigen (PCNA)-bind motif. **(B)** Decreased Cul3 in ASD patients can reduce the physical interaction between POLDIP1 and Cul3, resulting in an abnormal expression of RhoA, which subsequently affects fetal brain development. **(C)** POLDIP1 can target adenylosuccinate synthase (ADSS), and it can accelerate its ubiquitination and degradation. POLDIP1 will promote the degradation and reduction of ADSS, and that will promote carcinogenesis.

Nucleic POLDIP1 contains one functional domain and one functional motif. Its N-terminal has a BTB/POZ domain (residues 41–138) that is involved in the ubiquitination and degradation of ras homolog family member A (RhoA). The C-terminal possesses the motif QTKV-EFP (residues 249–255), a proliferating cell nuclear antigen (PCNA)-bind motif (Figure 1A) (1, 3). Co-induced with interleukin-6 (IL-6) and tumor necrosis factor-α (TNF-α), POLDIP1 can directly interact with PCNA and the small subunit (p50) of DNA polymerase δ to enhance DNA polymerase δ activity. Moreover, POLDIP1 can interact with a variety of proteins to play various biological functions (see Table 1).

**TABLE 1:** Binding partners, biological function and disease relevance of POLDIP1.

Binding partners	Type of mutation or deletion	Biological function	Disease relevance	Reference
Polymerase δ p50 subunit	NM	DNA replication and damage repair	NM	(1)
Proliferating cell nuclear antigen	NM	DNA replication and damage repair	NM	(1)
MYB proto-oncogene like 2 (MYBL2)	NM	DNA replication and damage repair	NM	(3)
Cullin-3 (Cul3)	NM	POLDIP1 and CUL3 work together to produce ubiquitin ligase complexes that specifically ubiquitinate ras homolog family member A (RhoA)	NM	(4)
NM	NM	RhoA levels rise when POLDIP1 is reduced, which inhibits synaptic transmission	POLDIP1 regulates the neuronal function relevant to neurogenesis, brain size and is involved in neuropsychiatric disorders.	(7)
NM	16p11.2 copy number variant (CNV)	POLDIP1 is a major driver for the neurodevelopmental phenotypes associated with the 16p11.2 CNV	16p11.2 CNV has been associated significantly and reproducibly with a range of neurocognitive defects, including epilepsy, autism and autism spectrum disorders (ASD)	(8, 12)
Cul3	NM	Non-sense mutations in Cul3 prevent POLDIP1 and Cul3 proteins from physically interacting	Psychiatric disorders-autism and schizophrenia	(9)
Rho family GTPase 2 and Rho family GTPase 3 (RND2 AND RND3)	NM	The long-term placement of cortical neurons throughout the postnatal mouse cerebral cortex is hampered by POLDIP1 expression disruptions. The branching and dendritic spine characteristics of layer II/III projection neurons are changed by forced expression of POLDIP1	The neurodevelopmental functions of POLDIP1 are likely to be relevant to human brain development and disease.	(10)
NM	16p11.2 deletion	A smaller 118 kb deletion within the recurrent 16p11.2 copy number variant (CNV) confers susceptibility to ASD and 5 out of the 27 genes are identified in the 16p11.2 deletion: major vault protein (MVP), CDP-diacylglycerol–inositol 3-phosphatidyltransferase (CDIPT), seizure related 6 homolog like 2 (SEZ6L2), aspartate beta-hydroxylase domain containing 1 (ASPHD1), and POLDIP1	Autism spectrum disorders (ASDs)	(11)
NM	NM	Adenylosuccinate synthetase (ADSS) can be targeted by POLDIP1 for ubiquitination and destruction	Lung cancer	(12)
NM	NM	POLDIP1 may play a beneficial effect in breast tumors, according to data extrapolated from the COSMIC database	Breast cancer	(14)

NM, no mention.

**FIGURE 2:**
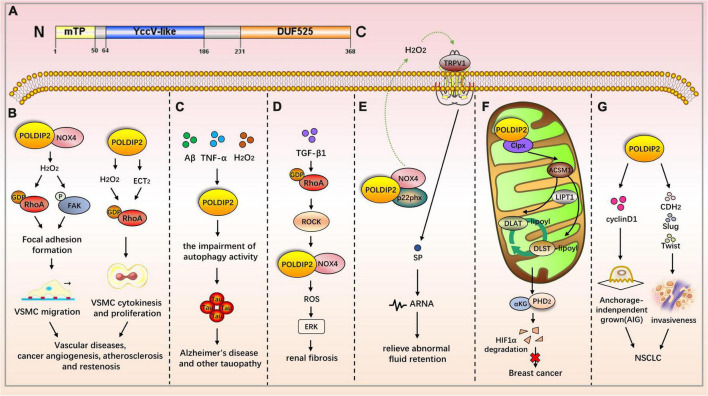
The role of POLDIP2 in the modulation of disease-related signaling pathways. **(A)** Domain map of POLDIP2. POLDIP2 contains an N-terminal mitochondrial targeting peptide (mTP) and two main functional domains: a DUF525 domain and a hemimethylated YccV-like domain. DUF525 may be involved in protein-protein interaction and cation efflux, respectively. YccV may bind to DNA and regulate its expression. **(B)** POLDIP2 can increase the focal adhesion turnover and cell polarization of VSMCs. POLDIP2 participates in the migration of VSMCs by promoting the NOX4/RhoA/FAK pathway. POLDIP2 is involved in the cytokinesis and proliferation of VSMCs by activating RhoGEF epithelial cell transformation sequence 2 (ECT2) and its downstream molecule RhoA. **(C)** POLDIP2 is a novel regulator of Tau aggregation in Alzheimer’s disease and another tauopathy. Expression of POLDIP2 can be increased in neuronal cells by the multiple stresses, including Aβ, TNF-α, and H_2_O_2_. POLDIP2 overexpression can induce impairments of autophagy activity and partially proteasome activity and subsequently result in Tau aggregation. **(D)** In the TGF-β-induced rat renal myofibroblast differentiation model, the activation of the RhoA/Rock/POLDIP2/NOX4/ROS pathway can induce the activation of renal myofibroblasts, which may be of great significance in the pathogenesis of renal fibrosis. **(E)** Lin et al. have studied the effect of NOX4/p22phox/POLDIP2 interactions on the activity of TRPV1-mediated mechanosensation. Using co-immunoprecipitation experiments, they have demonstrated that POLDIP2 can interact with NOX4 and p22phox in renal pelvis lysates. The elevation of intrapelvic pressure (IPP) stimulates POLDIP2 expression and enhances the association between POLDIP2 and p22phox, which further activates NOX4. NOX4 stimulates TRPV1 by producing H2O2, increases the release of substrate P (SP), and boosts afferent renal nerve activity (ARNA). The mechanism clarifies the cause of renal non-response to fluid retention and provides a new therapeutic strategy for relieving abnormal fluid retention. **(F)** POLDIP2, a mitochondrial protein, can increase mitochondrial lipoacylation, enhance cell respiration, and reduce the growth rate of cancer cells. POLDIP2 binding to CLPX can restrain lipoic acid-activating enzyme Ac-CoA synthetase medium-chain family member 1 (ACSM1) degradation. Consequently, lipoyl-AMP is produced, and lipoyltransferase 1 (LIPT1) has a substrate for the lipoylation of dihydrolipoamide S-acetyltransferase (DLAT) and dihydrolipoamide S-succinyltransferase (DLST). The lipoylation promotes the tricarboxylic acid (TCA) cycle, leading to prolyl hydroxylase-2 (PHD2) production and HIF-1α degradation. However, POLDIP2 expression is declined in breast cancer cells. **(G)** POLDIP2 is downregulated in NSCLC tissues, and the overexpressed POLDIP2 increases the anchorage independent growth (AIG) and proliferation of NSCLC cells. As shown in the mechanism study, POLDIP2 knockdown can significantly impair the expression of cell proliferation, cyclin D1, epithelial mesenchymal transition (EMT) markers, cdh2, and slug and twist, thereby indicating that POLDIP2 participates in regulating tumor growth and invasiveness.

Polymerase δ-interacting protein 2 is a mitochondrial protein with multiple subcellular localizations. POLDIP2 can be expressed in the mitochondria, nucleus, cell membrane, and mitotic spindle. POLDIP2 has two independent domains: the YccV domain at the N-terminal (residues 64–186) and the DUF525 domain at the C-terminal (residues 231–368) (Figure 2A) (4, 5). An assessment of the POLDIP2 sequence revealed three supposed PCNA binding motifs between residues 81–88, 151–158, and 193–200 (6). Ever-growing evidence has shown that, in addition to the interaction between PCNA and polymerase δ, POLDIP2 can interact with a variety of binding partners and participate in the cell cycle, focal adhesion transition, and cell migration (see Table 2).

**TABLE 2:** Binding partners, biological function and disease relevance of POLDIP2.

Binding partners	Type of mutation or deletion	Biological function	Disease relevance	Reference
Polymerase δ p50 subunit	NM	DNA replication and damage repair	NM	(6)
Proliferating cell nuclear antigen	NM	DNA replication and damage repair	NM	(6)
Polymerase η	NM	Translesion DNA synthesis (TLS)	NM	(20)
Polymerase η	NM	Translesion DNA synthesis (TLS)	NM	(20)
Rev1 polymerase	NM	Translesion DNA synthesis (TLS)	NM	(20)
Rev7 (Rev7p)	NM	Translesion DNA synthesis (TLS)	NM	(20)
Polymerase λ	NM	Translesion DNA synthesis (TLS)	NM	(20)
PrimPol	NM	Translesion DNA synthesis (TLS)	NM	(22)
HPV 16 E7	NM	Viral DNA replication	NM	(23)
p22phox (p22phox protein)	NM	POLDIP2 associates with p22phox to activate Nox4, leading to regulation of focal adhesion turnover and vascular smooth muscle cell (VSMC) migration, thus linking reactive oxygen species production and cytoskeletal remodeling.	Restenosis and atherosclerosis	(15)
p22phox OR NADPH oxidase 4 (Nox4)	NM	To activate Nox4 and produce H2O2, POLDIP2 joins with p22phox. Nox4 then activates transient receptor potential cation channel subfamily V member 1 (TRPV1), increasing the release of SP into the pelvic cavity.	Uroschesis	(24)
CEA cell adhesion molecule 1 (CEACAM1)	NM	POLDIP2 is translocated to spliceosomes in response to UV-induced DNA damage, where it aids in the alternative splicing of the MDM2 (MDM2 proto-oncogene) transcripts.	Diseases related to UV-induced DNA damage, such as cancers.	(25)
NM	NM	POLDIP2+/mice’s isolated aortas showed impaired potassium chloride and phenylephrine-induced contractions, increased stiffness, and decreased compliance, which were linked to the disruption of elastic lamellae and excessive extracellular matrix deposition.	Vascular diseases	(26)
NM	NM	POLDIP2 regulates the turnover of focal adhesions and force polarization to govern vascular smooth muscle cell migration in a Nox4/RhoA/FAK-dependent manner.	Arthritis, cancer, restenosis and atherosclerosis.	(27)
NM	NM	POLDIP2 is a novel regulator of Ect2 (the RhoGEF epithelial cell transforming sequence 2), and both proteins play a critical role in VSMC proliferation.	Vascular diseases, such as, atherosclerosis and restenosis	(28)
NM	NM	POLDIP2 is upregulated following ischemic stroke and mediates the breakdown of the blood–brain barrier (BBB) by boosting cerebral cytokine production and MMP activation.	Cerebral edema in the ischemic brain	(29)
NM	NM	Aβ-induced expression of POLDIP2 plays a crucial role in Tau aggregation via the impairment of autophagy activity.	Alzheimer’s disease and other tauopathy	(30)
NM	NM	The activation of kidney myofibroblasts by TGF-1 is mediated through RhoA/ROCK-dependent regulation of POLDIP2/Nox4.	Renal fibrosis	(31)
NM	NM	Poldip2 is involved in β2-integrin activation during the inflammatory response, which in turn mediates neutrophil-to-endothelium adhesion in lipopolysaccharide-induced acute respiratory distress syndrome.	Acute respiratory distress syndrome (ARDS)	(32)
Amplification of the genomic region on 17q11.2	NM	The TNFAIP1/POLDIP2 CSAGA is a clinically relevant transcriptional structural-functional gene module linked to erb-b2 receptor tyrosine kinase 2 (ERBB2) amplicon core gene expression in breast cancer and connected with amplification of the genomic region on 17q11.2.	Breast cancer	(33)
NM	NM	POLDIP2 is an oxygen-sensitive protein that controls PDH and alpha-ketoglutarate dehydrogenase subunit E2 (αKGDH) lipoylation and activation by a mechanism that involves regulation of the caseinolytic peptidase (Clp)-protease complex and production of the lipoate-activating enzyme Ac-CoA synthetase medium-chain family member 1 (ACSM1).	Cancer	(34)
NM	NM	In our study, 187 patients with NSCLC and 310 age- and gender-matched controls were included, as well as an independent set of 29 patients for validation. Dual specificity phosphatase 6 (DUSP6), EIF2S3 eukaryotic translation initiation factor 2 subunit gamma (eukaryotic translation initiation factor 2 subunit gamma), growth factor receptor bound protein 2 (GRB2), MDM2 proto-oncogene (MDM2), neurofibromin 1 (NF1), POLDIP2, ring finger protein 4 (RNF4), and WEE1 were identified as significant NSC (WEE1 G2 checkpoint kinase).	Non-small cell lung cancer	(35)
NM	NM	POLDIP2 gene functioned as an oncogene in NSCLC, implying that the oncogenic ability could be via cell proliferation or epithelial mesenchymal transition (EMT).	Non-small cell lung cancer	(36)
NM	Frameshift insertion (c.83dupG)	NM	HPV-negative undifferentiated tongue sarcoma	(37)
NM	Shear mutation of exon 1 (c.81 + 1 A > G)	NM	Secretory breast carcinoma (SBC)	(38)

NM, no mention.

**FIGURE 3:**
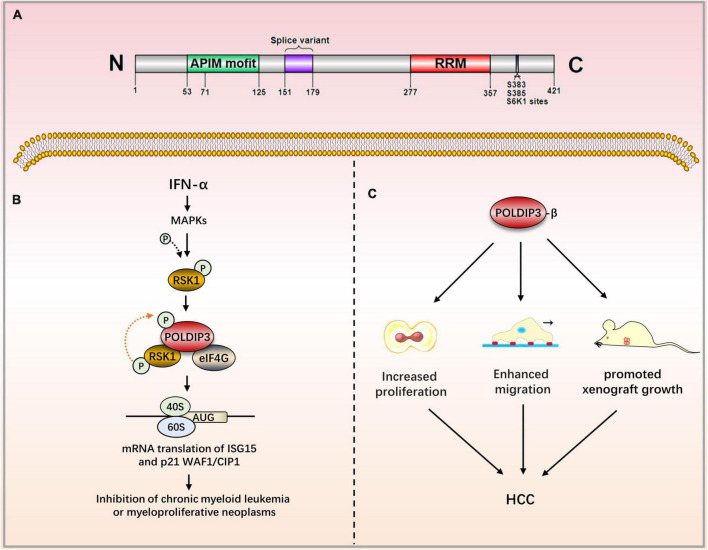
The role of POLDIP3 in the modulation of disease-related signaling pathways. **(A)** Domain map of POLDIP3. The N-terminal of POLDIP3 contains a cluster of five AlkB homolog 2 PCNA-Interacting motifs (APIMs), in which POLDIP3 can bind with PCNA and the p50 subunit of polδ at its residues 71–125 and 53–125, respectively. The C-terminal 277–357 amino acid residues of POLDIP3 possess an RRM similar to ALY/REF (RNA and export factor binding proteins) and can bind with RNA. Meanwhile, the RRM domain can bind with ribosomal protein S6 kinase 1 (S6K1). The phosphorylation of POLDIP3 locates at s383/s385. **(B)** Ribosomal protein S6 kinase (RSK1) can induce POLDIP3 phosphorylation in an IFN-α-dependent manner. POLDIP3 phosphorylation leads to greater interactions between POLDIP3 and eukaryotic initiation factor 4G (EIF4G) to form a unique IFN-induced RSK1-POLDIP3-EIF4G complex, thus promoting the mRNA translation of interferon-stimulated genes (ISG). **(C)** POLDIP3-β overexpression can increase the proliferation and migration of hepatocellular carcinoma cells and promote the growth of xenotransplantation. IFN-α, Type I interferons Alpha; RSK1, p90 ribosomal protein S6 kinase; eIF4G, eukaryotic initiation factor; ISG, IFN-stimulated genes; CML, chronic myeloid leukemia; HCC, Hepatocellular carcinoma.

The subcellular localization of POLDIP3 is mainly located in nuclear spots and at the exon junction complex (EJC). In mitotic cells, POLDIP3 is located in the cytoplasm. POLDIP3 also has two independent domains: the APIM domain (residues 53–125) and an RNA recognition motif (RRM) domain (residues 277–357) (Figure 3A) (7). POLDIP3 can interact with DNA polymerase δ and PCNA, and it plays an important role in DNA replication. POLDIP3 also has a variety of binding partners as well as many functions, several of which seem to be the result of specifically related protein interactions (see Table 3). Additionally, since POLDIP3 possesses RRM, it is speculated that POLDIP3 may participate in RNA processing, nuclear output, and stability to participate in RNA metabolism.

**TABLE 3:** Binding partners, biological function and disease relevance of POLDIP3.

Binding partners	Type of mutation or deletion	Biological function	Disease relevance	Reference
Polymerase δ p50 subunit	NM	DNA replication and damage repair	NM	(5)
Proliferating cell nuclear antigen	NM	DNA replication and damage repair	NM	(42)
Polymerase δ (Pol δ)	NM	POLDIP3 expression changes or mutations may affect Pol functions *in vivo* and thus be a nexus for altered genomic stability.	NM	(41)
RTEL1 (regulator of telomere elongation helicase 1)	NM	Loss of POLDIP3 and RTEL1 causes R-loop accumulation confined to active replication sites, which increases endogenous replication stress and fuels ensuing genomic instability.	Cancers	(43)
S6K1	NM	POLDIP3, which is deposited at the EJC during splicing, recruits S6K1 to newly synthesized mRNA, and SKAR and S6K1 increase the translation efficiency of spliced mRNA.	NM	(39)
Enhancer of rudimentary (ERH)	NM	Human enhancer of rudimentary (ERH) is a molecular partner of POLDIP3, a protein that regulates cell growth by interacting with DNA polymerase delta and S6K1.	NM	(42)
NM	NM	POLDIP3 co-IPs with TRanscription and EXport (TREX) components such as THO complex 2 (THOC2), UAP56 (ATP-dependent RNA helicase Uap56), and Aly (aly protein), and overexpression of POLDIP3, a new member of TREX, can cause massive mRNA exportation from the nucleus to be blocked.	NM	(45)
NM	NM	Ribosomal protein S6 kinase (RSK1) can phosphorylate POLDIP3 in an IFN-dependent manner. POLDIP3 phosphorylation increases its interaction with eukaryotic initiation factor 4G (EIF4G), resulting in the formation of a unique IFN-induced RSK1–POLDIP3-eif4g complex, which promotes mRNA translation of interferon-stimulated genes (ISG).	Leukemic neoplastic	(46)
NM	Wild-type POLDIP3 (variant-1) decreased, POLDIP3 lacking exon 3 (variant-2)	A remarkable splicing change in the polymerase delta interacting protein 3 (POLDIP3) as a result of TAR DNA binding protein (TDP-43) depletion in two types of cultured cells POLDIP3 (variant-1) decreased and POLDIP3 lacking exon 3 (variant-2) increased in cells treated with TDP-43 siRNA. POLDIP3 variant-2 mRNA was found to be increased in motor cortex, spinal cord, and spinal motor neurons collected by laser capture microdissection in patients with ALS.	Amyotrophic lateral sclerosis (ALS)	(47)
NM	NM	POLDIP3 as a new autoantigen that could be used to diagnose immune diseases.	Systemic vasculitis	(48)
NM	NM	POLDIP3 can be used as a gene associated with gastric cancer protection and as part of a prognosis model composed of 10 genes to predict treatment response and prognosis in patients with gastric cancer.	Gastric cancer	(49)
NM	Normal POLDIP3 (POLDIP3-α) lacks exon 3 and 29 amine acids (POLDIP3-β)	POLDIP3-(POLDIP3 transcript lacking exon 3 and 29 amino acids) expression was significantly increased in liver cancer tissues compared to paired adjacent normal liver tissues. POLDIP3-overexpression significantly increased the proliferation and migration of HCC cells and promoted the growth of xenotransplantation in *in vitro* and *in vivo* functional experiments. POLDIP3-will be a potential target for the treatment of liver cancer.	Hepatocellular carcinoma (HCC)	(50)
NM	NM	Clinical outcome association analysis revealed a significant connection between POLDIP3 expression and overall and relapse-free survival in neuroblastoma patients.	High-risk neuroblastoma	(51)

NM, no mention.

Although the POLDIP family has been linked to several neurological diseases, cancers, and other diseases, there may be many undiscovered links between the POLDIP family and other diseases, particularly tumors. While summarizing the relationship between the POLDIP family and a variety of diseases, this review also examined the relationships between POLDIP family protein expression and carcinogenesis, as well as the survival time of various types of tumors, using the TCGA database.

This review summarizes the POLDIP family’s involvement in various biological functions, as well as the pathways in which it participates during the occurrence of multiple diseases. This knowledge will help scientists find novel targets and treatment options for related diseases.

## Tissue expression and subcellular localization of polymerase δ-interacting protein 1

DNA POLDIP1, also known as BACURD1, FKSG86, PDIP1, hBACURD1, and KCTD13, was originally discovered by He et al. Through a yeast two-hybrid system experiment in 2000, He et al. (1) found a new protein interacting with small subunit p50 of DNA polymerase δ and named it POLDIP1. The human POLDIP1 gene is located on 16p11.2. Its molecular weight is about 30 kDa. POLDIP1 is widely expressed in multiple tissues, including the heart, kidney, and brain tissues.

Examining the subcellular distribution of POLDIP1 by indirect immunofluorescence and confocal microscopy, He et al. (1) found that POLDIP1 was located in the nucleus. Further studies found that POLDIP1 and PCNA were co-located in the replication focus of S-phase MCF-7 cells. Two conserved sites were found in the protein structure of the POLDIP1 gene: a BTB/POZ domain in the C-terminal region as well as a PCNA-binding motif in the N-terminal region (1, 2).

## The biological function of polymerase δ-interacting protein 1

Human POLDIP1 can interact with PCNA and p50, the small subunit of DNA polymerase δ; therefore, it can enhance the activity of DNA polymerase δ in the presence of PCNA. Its synthesis can be co-induced by TNF-α and IL-6 (1). POLDIP1, acting as a meson, can bind to MYB proto-oncogenes like 2 (MYBL2) and PCNA, thereby participating in the process of DNA repair (3). Utilizing a BTB/POZ domain with about 120 amino acid residues at its N-terminal, POLDIP1 can act as a substrate-specific adaptor protein to form a complex with cullin-3 ubiquitin ligase. POLDIP1 acts as an adaptor protein and participates in the ubiquitination and degradation of RhoA; it does so to maintain the actin cytoskeleton structure and cell morphology (4), promote the transmission of neural synapses, and to facilitate cell migration as well as the development of brain size (7–9). POLDIP1 is an interaction partner of Rnd2 and Rnd3, both of which are members of the rho family GTPase (RND) protein family. This interaction affects the long-term localization and dendritic maturation of cortical neurons (10).

### Polymerase δ-interacting protein 1 and nervous system diseases

Polymerase δ-interacting protein 1 plays an important role in neurocognitive impairment. The POLDIP1 gene can modulate early neural development and the head size phenotype. Copy number deletion of POLDIP1 (located at 16p11.2) led to the macrocephalic phenotype, whereas copy number duplication of POLDIP1 yielded a microcephalic phenotype in zebrafish embryos (8). The integration of zebrafish and mouse data has indicated that the microcephalic phenotype results from decreased neural progenitor cells and increased apoptosis of developing brain cells, whereas the macrocephalic phenotype is attributed to increased neural progenitor cells and no alteration in brain cell apoptosis. Some studies have also demonstrated that POLDIP1 knockout reduced the synaptic transmission in area CA1 of the hippocampus, although they have proven that POLDIP1 deletion cannot affect brain size (7). A critical 118 kb-deletion region on 16p11.2 from a three-generation autism spectrum disorder (ASD) family has been identified, and POLDIP1 is located exactly within the region (11). Meanwhile, researchers have revealed copy number variants (CNVs) in a family pedigree of autism on 16p11.2, in which they have analyzed both inherited and *de novo* rearrangements of POLDIP1 (8). POLDIP1 is a target gene that causes cognitive dysfunction in patients with 16p11.2 deletion (12). Decreased Cul3 in ASD patients can reduce the physical interaction between POLDIP1 and Cul3, resulting in an abnormal expression of RhoA, which subsequently affects fetal brain development (9).

### Polymerase δ-interacting protein 1 and cancers

Polymerase δ-interacting protein 1 has recently been implicated in biological ubiquitination and protein degradation. POLDIP1 can bind to and accelerate the ubiquitination and degradation of adenylosuccinate synthase (ADSS), an enzyme that catalyzes the synthesis of adenosine monophosphate (AMP). ADSS expression in lung adenocarcinoma tissues is significantly lower than in normal tissues, according to researchers. Based on the above findings, it is hypothesized that POLDIP1 will promote the degradation and reduction of ADSS and that it will promote carcinogenesis (13). Furthermore, data from the cosmic database show that POLDIP1 may promote the occurrence of breast tumors, as high expression of POLDIP1 has been found in 14% of breast tumor samples (14).

## Tissue expression and subcellular localization of polymerase δ-interacting protein 2

Liu et al. (6) identified a novel protein that can bind to the p50 subunit of DNA polymerase delta. They named it PDIP38, which is an alias for DNA POLDIP2. POLDIP2 is also known as pold4, pdip38, and p38. The human POLDIP2 gene is located at 17q11.2, and it encodes a protein with a molecular weight of about 28 kDa. POLDIP2 is generally expressed in 27 tissues, including kidney, liver, and heart tissues among others. Of note, the expression level of POLDIP2 in myeloid cells is very low (15).

Polymerase δ-interacting protein 2 has been found in multiple organelles, including mitochondria, spliceosomes, and nuclei. Its subcellular localization depends on the state of cell proliferation and its interactions with cell adhesion receptors. Cell fractionation experiments have shown that the majority of POLDIP2 can distribute in mitochondrial precipitation and that only a very small amount of it is present in the nucleus (16). However, there are disagreements about the subcellular localization of POLDIP2. POLDIP2 is dynamically localized in the cell surface or the nucleus under the influence of carcinoembryonic antigen-related cell adhesion molecule 1 (CEACAM1, CD66a), which acts as a cell adhesion receptor as well as an interacting protein of POLDIP2 (17). The above results indicate that POLDIP2 can regulate multiple cellular functions based on its subcellular localization and binding partners (17, 18).

## The biological function of polymerase δ-interacting protein 2

Liu et al. (6) identified POLDIP2 as a binding chaperone of p50 (a small subunit of DNA polymerase δ). POLDIP2 is a multi-functional protein dealing in DNA replication and repair. In addition to its role in DNA replication and damage repair, POLDIP2 is involved in mitochondrial function modulation, extracellular matrix (ECM) regulation, cell cycle progression, focal adhesion turnover, and cell migration (19). In addition to p50, data have indicated that POLDIP2 can interact with a variety of proteins and perform its multiple functions. By binding to polymerase δ and PCNA, POLDIP2 can participate in DNA replication (6). Additionally, POLDIP2, as an intermediate, interacts with Pol η, Polζ, and Rev1, and it is involved in DNA replication and DNA translesion synthesis (TLS) (20). When 8-oxo-7,8-dihydroguanine (8-oxo-G) lesions occur during DNA replication, POLDIP2 can interact with Pol λ, which is involved in the correct bypass of 8-oxo-7,8-dihydroguanine (8-oxo-G) lesions (21). POLDIP2 can also stimulate the activity of primpol and therefore enhance primpol’s ability to bind DNA, which plays a repair role in 8-oxo-G TLS damage (22). Interacting with the E7 oncoprotein of human papillomavirus 16 and simultaneously inhibiting Pol δ activity, POLDIP2 plays an important role in Pol δ-mediated viral DNA replication (23). Binding with p22phox to stimulate NADPH oxidase 4 (NOX4), POLDIP2 controls focal adhesion turnover and affects vascular smooth muscle cell (VSMC) migration (15). POLDIP2 can interact with NOX4 or P22phox, and it can regulate the activity of the transient receptor potential vanilloid 1 (TRPV1) channel in rat kidneys (24). After stimulating NOX4, POLDIP2 expression increases the production of endogenous reactive oxygen species (ROS) catalyzed by NOX4, thereby indicating its crucial role in regulating the nuclear redox environment (15). POLDIP2 can interact with CEACAM1 (carcinoembryonic antigen-related cell adhesion molecule 1, CD66a) to manage its subcellular localization, which in turn facilitates CEACAM1-mediated cell survival, differentiation, and growth (17). Following UV-induced DNA damage, POLDIP2 translocates to the spliceosome loci of mouse double minute 2 homolog (MDM2). In doing so, it participates in UV-induced selective MDM2 transcript splicing (25).

### Polymerase δ-interacting protein 2 and cardiovascular disease

Defective POLDIP2 can decrease the activity of NOX4 in cardiac muscle tissues. POLDIP2 knockout promotes the destruction of the aortic valve’s elastic lamina. This leads to excessive deposition of the ECM, resulting in impaired aortic contractions and reduced compliance. Further mechanism studies have indicated that defective POLDIP2 decreases NOX4 activity; the VSMCs consequently produce less ROS and secrete more type I collagen. This indicates that POLDIP2 is involved in regulating NADPH oxidase activity, thus affecting the structure and function of vessels (26). POLDIP2 can increase the focal adhesion turnover and cell polarization of VSMCs. POLDIP2 participates in the migration of VSMCs by promoting the NOX4/RhoA/FAK pathway (27). The excessive proliferation of VSMCs is one mechanism of atherosclerosis and restenosis. POLDIP2 is involved in the cytokinesis and proliferation of VSMCs by activating RhoGEF epithelial cell transformation sequence 2 (ECT2) and its downstream molecule RhoA (28).

### Polymerase δ-interacting protein 2 and neurological diseases

Polymerase δ-interacting protein 2 is upregulated after ischemic stroke, which boosts cytokine TNF-α and IL-6 production as well as matrix metalloproteinase (MMP) activation, thereby mediating the increase of blood–brain barrier (BBB) permeability. Therefore, POLDIP2 is a potential drug target to reduce edema and stroke mortality (29). Neuronal degeneration caused by Tau aggregation can be found in a variety of neurological diseases, including Alzheimer’s disease and Parkinson’s disease. Studies have shown that POLDIP2 regulates Tau aggregation. POLDIP2 expression increases Tau aggregation without affecting Tau phosphorylation. It has been found that POLDIP2 overexpression can result in reduced cellular autophagy activity and partial proteasome activity, which are primarily performed by the DUF525 domain of POLDIP2 (30).

### Polymerase δ-interacting protein 2 and renal diseases

In the TGF-β-induced rat renal myofibroblast differentiation model, the activation of the RhoA/Rock/POLDIP2/NOX4/ROS pathway can induce the activation of renal myofibroblasts, which may be of great significance in the pathogenesis of renal fibrosis (31). However, POLDIP2 controls VSMC migration by activating NOX4/RhoA (27). The activation pathways mediated by POLDIP2 in the two kinds of literature are different. Lin et al. have studied the effect of NOX4/p22phox/POLDIP2 interactions on the activity of TRPV1-mediated mechanosensation. Using co-immunoprecipitation experiments, they have demonstrated that POLDIP2 can interact with NOX4 and p22phox in renal pelvis lysates. The elevation of intrapelvic pressure (IPP) stimulates POLDIP2 expression and enhances the association between POLDIP2 and p22phox, which further activates NOX4. NOX4 stimulates TRPV1 by producing H2O2, increases the release of substrate P (SP), and boosts afferent renal nerve activity (ARNA). The effect is to make the body respond to the changes in hydrostatic pressure in the renal pelvis. The mechanism clarifies the cause of renal non-response to fluid retention and provides a new therapeutic strategy for relieving abnormal fluid retention (24).

### Polymerase δ-interacting protein 2 and respiratory disease

Acute respiratory distress syndrome (ARDS) is a fatal disease marked by acute hypoxia and non-cardiogenic pulmonary edema. Poldip2 is involved in the activation of β_2_-integrin during the inflammatory response, which mediates neutrophil-to-endothelium adhesion in ARDS (32).

### Polymerase δ-interacting protein 2 and cancers

Grinchuk et al. have identified a new complex sense-antisense architecture (CSAGA) on 17q11. The CSAGA contains five genes: tmem97, ift20, tnfaip1, polip2, and tmem199, all of which are termed the tnfaip1/POLDIP2 CSAGA. This CSAGA is linked to the amplification of 17q11.2 genomic regions and is associated with the expression of ERBB2 in breast cancer. The co-expression pattern of the CSAGA may correlate to the histological grade and prognosis of breast cancer (33). Downregulation of POLDIP2 and binding to CLPX allow for caseinolytic peptidase (Clp) activation and lipoic acid-activating enzyme Ac-CoA synthetase medium-chain family member 1 (ACSM1) degradation. Consequently, no lipoyl-AMP is produced, and lipoyltransferase 1 (LIPT1) has no substrate for the lipoylation of dihydrolipoamide S-acetyltransferase (DLAT) and dihydrolipoamide S-succinyltransferase (DLST). The lipoylation defect represses the tricarboxylic acid (TCA) cycle, leading to HIF-1α prolyl hydroxylase-2 (PHD2) metabolic inhibition and HIF-1α lasting expression. Overexpression of POLDIP2, a mitochondrial protein, can increase mitochondrial lipoacylation, enhance cell respiration, and reduce the growth rate of cancer cells, thereby indicating the key role of POLDIP2 in hypoxia and metabolic adaptation of breast cancer cells (34). Gene analyses from peripheral blood mononuclear cells have shown that POLDIP2 mRNA expression was negatively correlated with the risk of non-small cell lung cancer (NSCLC) (35). POLDIP2 is downregulated in NSCLC tissues, and the overexpressed POLDIP2 increases the anchorage independent growth (AIG) and proliferation of NSCLC cells. As shown in the mechanism study, POLDIP2 knockdown can significantly impair the expression of cell proliferation, cyclin D1, epithelial mesenchymal transition (EMT) markers, cdh2, and slug and twist, thereby indicating that POLDIP2 participates in regulating tumor growth and invasiveness (36). Additionally, POLDIP2 mutations have been linked to the development of tumors. A frameshift mutation in POLDIP2 was discovered in HPV-negative undifferentiated tongue sarcoma (37). Shear mutation of POLDIP2 was found in secretory breast carcinoma (SBC) (38).

## Tissue expression and subcellular localization of polymerase δ-interacting protein 3

Polymerase δ-interacting protein 3, also known as pdip3, pdip46, and Skar, was identified by Hernandes et al. in 2003. Through a yeast two-hybrid experiment, they found that POLDIP3, like POLDIP2, was a novel protein interacting with the p50 small subunit of human DNA polymerase δ (pol δ) (6). The human POLDIP3 gene is located on chromosome 22q13.2. The total molecular weight of POLDIP3 is about 42 kD. Human POLDIP3 is widely expressed in 27 tissues, including the spleen, ovary, and bone marrow tissues. The POLDIP3 protein is mainly located in the nucleus and at the EJC (39). In mitotic cells, POLDIP3 is located in the cytoplasm (40). The N-terminal of POLDIP3 contains a cluster of five AlkB homolog 2 PCNA-Interacting motifs (APIMs), in which POLDIP3 can bind with PCNA and the p50 subunit of pol δ at its residues 71–125 and 53–125, respectively (41). The C-terminal 277–357 amino acid residues of POLDIP3 possess an RRM similar to ALY/REF (RNA and export factor binding proteins) and can bind with RNA (39). Meanwhile, the RRM domain can bind with ribosomal protein S6 kinase 1 (S6K1), as well (41).

## The biological functions of polymerase δ-interacting protein 3

Since the discovery of POLDIP3, little research focused on its function has been performed. POLDIP3 can interact with DNA Pol δ and PCNA to promote cell replication and proliferation (42). POLDIP3 can directly communicate with Pol δ to activate DNA polymerase δ. Therefore, the alteration of POLDIP3 expression or its mutation may affect the function of Pol δ, thereby modulating genomic stability (41). POLDIP3 plays a role in the synthesis of leading-strand and lagging-strand DNA. During lagging strand DNA synthesis, when Polδ3 encounters secondary structure obstacles, POLDIP3 will accelerate the dissociation of Polδ3 from DNA and trigger the conversion of DNA polymerase to Polδ4/POLDIP3. Similarly, Pol ε is a principal enzyme for leading strand DNA synthesis. When Pol ε faces secondary structure obstacles during DNA synthesis, POLDIP3 can boost the depolymerization of Pol ε from DNA and ignite the switch of DNA polymerase from Pol ε to Polδ4/POLDIP3 (16). The functional interaction between telomere length 1 (RTEL1) and POLDIP3 has been recently demonstrated. An R-loop (RNA-DNA hybridization) at the genomic region where transcription and replication intersect can obstruct DNA replication. When DNA replication is blocked, POLDIP3 acts as a subunit of Pol δ; With other components of PCNA, it can also recruit RETL1 to the replication fork of the stalled transcription site to prevent a collision between the replication fork and RNA polymerase.

This in turn prevents an R-loop; and finally, this protects genome-wide replication and genome integrity (43). POLDIP3 can interact with ribosomal protein S6K1 to enhance the translation efficiency of mRNA (40). Under the stress state, the activated mTOR signaling pathway can induce POLDIP3, which is already located on the EJC. This leads to the recruitment of S6K1 into the newly synthesized mRNA, thereby facilitating the phosphorylation of several proteins in a cap-binding complex-mRNA protein (CBC mRNP) complex. The effect is to promote the translation efficiency of mRNA (40). Meanwhile, activated by the mTOR and PI3K signaling pathways, S6K1 triggers the phosphorylation of POLDIP3 at s383/s385. This phosphorylation is necessary to combine S6K1 and POLDIP3 (39). Because of its RRM, POLDIP3 has been speculated to be involved in RNA processing, nuclear export, and stability. It also takes part in RNA metabolism (39).

Polymerase δ-interacting protein 3 is the binding partner of the enhancer of the rudimentary homolog (ERH), as well. ERH is a transcription regulator that affects the expression of several genes in the cell cycle. Further studies have found that the site of interaction with ERH is located at POLDIP3’s C-terminal residues 274–421. This region is not continuous and can be further divided into two sub-regions. The larger (sub-region I) contains residues 274–368, and the smaller (sub-region II) contains residues 379–421. Since POLDIP3 is thought to affect cell size, this interaction of ERH/POLDIP3 is widespread in cell growth control (42). Recently, POLDIP3 has been identified as a novel member of the human transcription-export (TREX) complex. It is well known that the TREX complex plays an important role in mRNA nuclear export (44). Moreover, it has been demonstrated that overexpression of POLDIP3 can lead to the obstruction of enormous mRNA exportation from the nucleus (45). Ribosomal protein S6 kinase (RSK1) can induce POLDIP3 phosphorylation in an IFN-α-dependent manner. POLDIP3 phosphorylation leads to greater interactions between POLDIP3 and eukaryotic initiation factor 4G (EIF4G) to form a unique IFN-induced RSK1-POLDIP3-EIF4G complex, thus promoting the mRNA translation of interferon-stimulated genes (ISG) (46).

### Polymerase δ-interacting protein 3 and neurological diseases

Studies have shown that the alternative splicing of POLDIP3 is related to amyotrophic lateral sclerosis (ALS). ALS is a neurodegenerative disease caused by the selective loss of motor neurons. In ALS motoneurons, the TDP-43 protein (43 kDa tar DNA binding protein) dislocates from the nucleus and enters the cytoplasm to form an inclusion body. Depletion of TDP-43 can result in abnormal splicing of POLDIP3 mRNA. In TDP-43 siRNA-treated cell models, wild-type POLDIP3 (variant 1) decreased, while mutated POLDIP3 (variant 2, lacking exon 3) increased. Therefore, detecting the variety law of POLDIP3/variant-2 in cerebrospinal fluid is of great significance for the diagnosis and evaluation of the progress of ALS (47).

### Polymerase δ-interacting protein 3 and immune diseases

Systemic vasculitis forms a group of heterogeneous autoimmune diseases characterized by vascular inflammation and an antibody reaction with autoantigens in the vascular wall. Using the serum of patients with clinical atypical renal vasculitis, researchers have found that POLDIP3 is a novel autoantigen, one that can be used as a diagnostic marker of autoimmune disease systemic vasculitis (48).

### Polymerase δ-interacting protein 3 and cancers

Kroczynska et al. have demonstrated that POLDIP3 plays an important role in type I interferons (IFN)-induced anti-leukemia and anti-tumor responses. Experiments have shown that POLDIP3 knockdown in primary leukemia CFU-GM progenitor cells from patients with chronic myeloid leukemia (CML)–or in primary malignant early erythrocyte progenitor cells (BFU-E) from patients with polycythemia vera–have reduced IFN-α-induced inhibitory effects on colony formation. Mechanism studies have illuminated how POLDIP3 knockdown results in the mRNA translation defects of key interferon stimulated genes (ISG) ISG15 and p21WAF1/CIP1 (41). Kroczynska et al. evaluated the effect of POLDIP3 knockdown on IFN-α-induced anti-colon adenocarcinoma responses. The results have shown that IFN-α treatment can inhibit the growth of HT29, a malignant colon adenocarcinoma cell; this inhibition, however, is reversed by POLDIP3 knockdown. This study established the role of POLDIP3 in IFN-α-induced colon cancer prevention (41). Lou et al. have constructed a prognosis model composed of 10 genes related to RNA processing, including POLDIP3, by using bioinformatics methods that predict the prognosis of patients with gastric cancer. They have found that their prognosis model could be used to predict the treatment response and prognosis of patients suffering from gastric cancer (49). Liu et al. also found that the expression of POLDIP3-β (POLDIP3 transcript lacking exon 3) in liver cancer tissues is significantly upregulated compared to paired adjacent non-cancerous liver tissues. POLDIP3-β overexpression can increase the proliferation and migration of hepatocellular carcinoma cells and promote the growth of xenotransplantation. POLDIP3-α (full-length containing exon 3) has much weaker effects on HCC cells. In short, POLDIP3-β will be a promising target for the treatment of liver cancer (50). Utilizing network-based microarray analysis and a visualization platform,^1^ researchers have found that a loss in POLDIP3 copy numbers leads to poor overall or recurrence-free survival in patients with neuroblastoma (51).

## Conclusion

To date, the DNA POLDIP family contains three members, all of which can interact with DNA polymerase δ. It is widely known that human Pol δ (DNA polymerase delta) was first isolated from bovine bone marrow by Byrnes in 1976. The Pol δ belongs to the B family and is one of the principal DNA polymerases in eukaryotes. DNA polymerase δ is a holoenzyme composed of p125, p50, p68, and p12 subunits. It plays an important role in leading-strand and lagging-strand DNA synthesis (1, 2). To further study the role of the Pol δ, researchers in different laboratories have used yeast two-hybrid technology to find its interacting proteins. As a result, the three POLDIPs binding to the p50 subunit of Pol δ were identified by two separate laboratories. Researchers initially believed that POLDIPs were mainly involved in DNA replication and damage repair. However, recent strecent have confirmed that POLDIPs are involved in the regulation of signal transduction pathways in neurodevelopment, neuropsychiatric diseases, cardiovascular diseases, tumors, and other diseases. However, each protein participates in different signaling pathways.

Polymerase δ-interacting protein 1 research has primarily focused on neural development. POLDIP1 is the primary determinant of head size, and it can regulate early neural development (see Figure 1). POLDIP1 can be used as a substrate-specific adaptor protein to form a complex with cullin-3 ubiquitin ligase, as well as to participate in the ubiquitination and degradation of a wide range of proteins (4, 11).

So far, research on POLDIP2 is the most extensive and comprehensive, focusing mainly on cardiovascular disease, neurological disease, renal disease, and cancers (Figure 2). After interacting with NOX4, POLDIP2 participates in the migration and proliferation of VSMCs and renal myofibroblasts, thus playing an important role in vascular and renal fibrosis-related diseases (27, 31). However, the mechanism of its involvement in neurological disease and tumors need to be illuminated.

Few studies on POLDIP3 have been performed to date. Relevant studies on POLDIP3 have mainly assessed its role in the occurrence of neurological diseases, immune diseases, and cancer. However, the precise mechanisms remain unclear (Figure 3).

**FIGURE 4:**
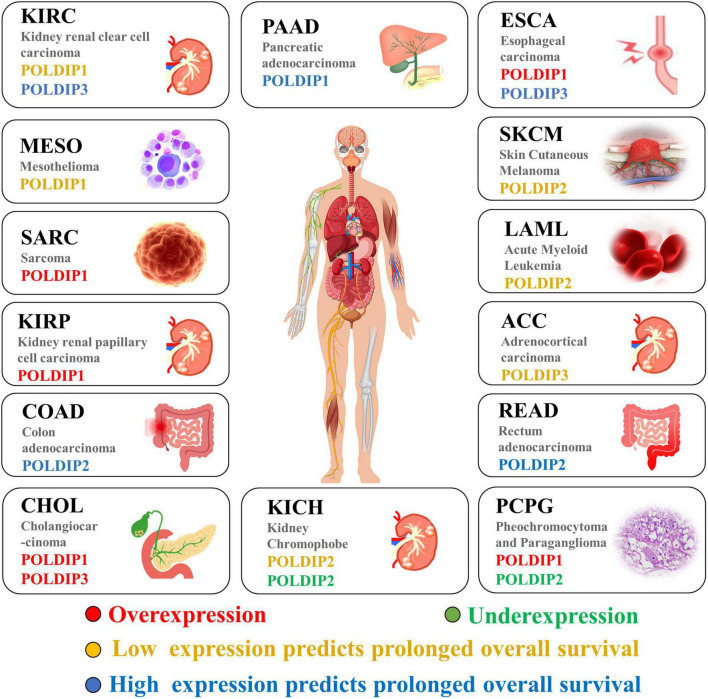
The POLDIP family in human cancers. Synthesis of the current knowledge on the role of POLDIP proteins in different cancer types. POLDIPs whose expression is downregulated in cancers are in green; POLDIPs whose expression is upregulated in cancers are in red; POLDIPs whose low-expression predicts prolonged overall survival are in yellow; POLDIPs whose high-expression predicts prolonged overall survival are in blue; all information is the result of data extrapolated by analysis of TCGA.

Last but not least, we examined and summarized the gene expressions of the POLDIP family members in a range of malignancies to offer fresh perspectives to the scientific community. Using the TCGA database, we also looked at the relationships between their expressions and overall patient survival times (summarized in Figure 4). For researchers working on cancer, our data summary can offer fresh perspectives.

Regarding the various therapeutic targeting options for the POLDIP family in diseases, therapeutic modalities of diseases will primarily focus on several approaches described below and in Table 4: (1) POLDIPs gene therapy, for instance, adenoviral-mediated POLDIPs overexpression/inhibition, (2) targeted the py against POLDIPs interactive proteins (3) targeted therapy against critical factors involved in POLDIPs-related signaling pathways, and (4) POLDIPs mutants-related therapy, that is to develop mutant-specific POLDIPs restoration drugs. However, many aspects of POLDIPs’ function, such as the details of POLDIPs’ interactive proteins and the precise knowledge of POLDIPs mutations in diseases, have remained elusive. POLDIPs targeting therapy is still in its early stages.

**TABLE 4:** The various possibilities for the therapeutic targeting of the POLDIPs in diseases.

Gene family members	Biological function and diseases	Disease relevance	Possible therapeutic strategies
**POLDIP1**	POLDIP1 reduction leads to increased RhoA levels that reduce synaptic transmission.	Neuronal POLDIP1 reduction is involved in neuropsychiatric disorders	Gene therapy based on adenoviral-mediated POLDIP1 overexpression et al.
	The neurodevelopmental abnormalities connected to the 16p11.2 Copy number variations are mostly driven by POLDIP1 (CNVs).	When 16p11.2 CNV deletion event occurs, it may lead to a decrease of POLDIP1 levels in patients with macrocephaly, epilepsy, ASD, et al. When 16p11.2 CNV duplication event occurs, it may lead to an increase of POLDIP1 levels in patients with microcephaly, autism.	Gene therapy based on CRISPR-Cas9 Gene Editing.
	ASD individuals with lower Cul3 levels may have less physical interaction between POLDIP1 and Cul3, which could lead to RhoA expression that is aberrant and thus disrupt fetal brain development.	Psychiatric disorders-autism and schizophrenia	Targeted therapy against critical factors involved in POLDIP1-related signaling pathways: adenoviral-mediated Cul3/RhoA overexpression et al.
	Adenylosuccinate synthase (ADSS) is a target of POLDIP1, which can hasten its ubiquitination and destruction. POLDIP1 will encourage ADSS decrease and degradation and, ultimately, encourage carcinogenesis.	Lung cancer	(1) Gene therapy based on adenoviral-mediated POLDIP1 inhibition, CRISPR-Cas9 Gene Editing et al. (2) Targeted therapy against POLDIP1 interactive proteins: adenoviral-mediated ADSS overexpression, et al.
	POLDIP1 may play a beneficial effect in breast tumors, according to data extrapolated from the COSMIC database.	Breast cancer	POLIDP1 gene therapy: adenoviral-mediated POLDIP1 inhibition, CRISPR-Cas9 Gene Editing, et al.
**POLDIP2**	POLDIP2 associates with p22phox to activate Nox4, leading to regulation of focal adhesion turnover and vascular smooth muscle cell (VSMC) migration, thus linking reactive oxygen species production and cytoskeletal remodeling.	Restenosis and atherosclerosis	(1) POLIDP2 gene therapy: adenoviral-mediated POLDIP2 overexpression et al. (2) Targeted therapy against POLDIP2 interactive proteins: adenoviral-mediated p22phox overexpression et al.
	To activate Nox4 and produce H2O2, POLDIP2 joins with p22phox. Nox4 then activates TRPV1 (transient receptor potential cation channel subfamily V member 1), increasing the release of SP into the pelvic cavity.	Uroschesis	(1) POLIDP2 gene therapy: adenoviral-mediated POLDIP2 overexpression et al. (2) Targeted therapy against POLDIP2 interactive proteins: adenoviral-mediated p22phox and Nox4 overexpression et al.
	POLDIP2+/mice’s isolated aortas showed impaired potassium chloride and phenylephrine-induced contractions, increased stiffness, and decreased compliance, which were linked to the disruption of elastic lamellae and excessive extracellular matrix deposition.	Vascular diseases	POLIDP2 gene therapy: adenoviral-mediated POLDIP2 overexpression et al.
	POLDIP2 controls vascular smooth muscle cell migration by regulating focal adhesion turnover and force polarization in a Nox4/RhoA/FAK-dependent manner.	Arthritis, cancer, restenosis and atherosclerosis.	Targeted therapy against critical factors involved in POLDIP2-related signaling pathways: adenoviral-mediated Nox4/RhoA/FAK overexpression et al.
	POLDIP2 regulates focal adhesion turnover and force polarization in a Nox4/RhoA/FAK-dependent way to control vascular smooth muscle cell migration.	Vascular diseases, such as, atherosclerosis and restenosis	Targeted therapy against critical factors involved in POLDIP2-related signaling pathways: adenoviral-mediated Ect2 overexpression et al.
	POLDIP2 is upregulated following ischemic stroke and mediates the breakdown of the blood–brain barrier (BBB) by increasing cerebral cytokine production and MMP activation.	Cerebral edema in the ischemic brain	POLIDP2 gene therapy: adenoviral-mediated POLDIP2 overexpression et al.
	Aβ-induced expression of POLDIP2 plays a crucial role in Tau aggregation via the impairment of autophagy activity.	Alzheimer’s disease and other tauopathy	Gene therapy based on adenoviral-mediated inhibition, CRISPR-Cas9 Gene Editing et al.
	The activation of kidney myofibroblasts by TGF-1 is mediated through RhoA/ROCK-dependent regulation of POLDIP2/Nox4.	Renal fibrosis	(1) Gene therapy: adenoviral-mediated POLDIP2 inhibition, CRISPR-Cas9 Gene Editing et al. (2) Targeted therapy against POLDIP2 interactive proteins: adenoviral-mediated Nox4 inhibition, CRISPR-Cas9 Gene Editing et al.
	POLDIP2 is an oxygen-sensitive protein that controls PDH and alpha-ketoglutarate dehydrogenase subunit E2 (αKGDH) lipoylation and activation by regulating of the caseinolytic peptidase (Clp)-protease complex and degradating the lipoate-activating enzyme Ac-CoA synthetase medium-chain family member 1 (ACSM1).	Cancer	Gene therapy: adenoviral-mediated POLDIP2 inhibition, CRISPR-Cas9 Gene Editing et al.
	187 patients with NSCLC and 310 age- and gender-matched controls, and an independent set containing 29 patients for validation were included. Eight significant NSCLC-associated genes were identified, including dual specificity phosphatase 6 (DUSP6), EIF2S3 eukaryotic translation initiation factor 2 subunit gamma (eukaryotic translation initiation factor 2 subunit gamma), growth factor receptor bound protein 2 (GRB2), MDM2 proto-oncogene (MDM2), neurofibromin 1 (NF1), POLDIP2, ring finger protein 4 (RNF4), and WEE1 (WEE1 G2 checkpoint kinase).	Non-small cell lung cancer	Gene therapy: adenoviral-mediated POLDIP2 inhibition, CRISPR-Cas9 Gene Editing et al.
	The POLDIP2 gene was found to function as an oncogene in NSCLC, suggesting that it may have the ability to cause cancer via stimulating cell proliferation or epithelial mesenchymal transition (EMT).	Non-small cell lung cancer	Gene therapy: adenoviral-mediated POLDIP2 inhibition, CRISPR-Cas9 Gene Editing et al.
	Poldip2 is involved in β2-integrin activation during the inflammatory response, which in turn mediates neutrophil-to-endothelium adhesion in lipopolysaccharide-induced acute respiratory distress syndrome.	Acute respiratory distress syndrome (ARDS)	Gene therapy based on adenoviral-mediated POLDIP2 inhibition, CRISPR-Cas9 Gene Editing et al.
	POLDIP2 mutants were found in HPV-negative undifferentiated tongue sarcoma.	Undifferentiated tongue sarcoma.	POLDIP2 gene therapy based on CRISPR-Cas9 Gene Editing et al.
	POLDIP2 mutants were found Secretory breast carcinoma (SBC).	Secretory breast carcinoma (SBC).	POLDIP2 gene therapy based on CRISPR-Cas9 Gene Editing et al.
**POLIDP3**	Loss of RTEL1 and POLDIP3 leads to R-loop accumulation that is confined to sites of active replication, enhances endogenous replication stress, and fuels ensuing genomic instability.	Cancers	(1) Gene therapy based on adenoviral-mediated POLIDP3 inhibition, CRISPR-Cas9 Gene Editing et al. (2) Targeted therapy against POLDIP3 interactive proteins: adenoviral-mediated RTEL1 overexpression et al.
	Ribosomal protein S6 kinase (RSK1) can induce POLDIP3 phosphorylation in an IFN-α-dependent manner. POLDIP3 phosphorylation leads to enhanced interaction between itself and eukaryotic initiation factor 4G (EIF4G), therefore forms a unique IFN-induced RSK1–POLDIP3-EIF4G complex, thus promoting the mRNA translation of interferon-stimulated genes (ISG).	Leukemic neoplastic	(1) Gene therapy based on adenoviral-mediated POLIDP3 inhibition, CRISPR-Cas9 Gene Editing et al. (2) Targeted therapy against POLDIP3 interactive proteins: Gene therapy based on adenoviral-mediated RSK1/EIF4G inhibition, CRISPR-Cas9 Gene Editing et al.
	In the cells treated with TAR DNA binding protein (TDP-43) siRNA, wild-type POLDIP3 (variant-1) decreased and POLDIP3 lacking exon 3 (variant-2) increased. An increment of POLDIP3 variant-2 mRNA have been found in motor cortex, spinal cord and spinal motor neurons collected by laser capture microdissection with ALS.	Amyotrophic lateral sclerosis (ALS)	(1) POLIDP3 gene therapy: adenoviral-mediated POLDIP3 (variant-1) overexpression. And POLDIP3 lacking exon 3 (variant-2) inhibition. (2) Targeted therapy against POLDIP3 interactive proteins: TDP-43 overexpression.
	POLDIP3 as new autoantigens that could be used as markers in the diagnosis of immune diseases.	Systemic vasculitis	Gene therapy based on adenoviral-mediated POLIDP3 inhibition, CRISPR-Cas9 Gene Editing et al.
	The expression of POLDIP3-β (POLDIP3 transcript lacking exon 3 and 29 amino acids) was significantly upregulated in liver cancer tissues compared with paired adjacent normal liver tissues. The functional experiments *in vitro* and *in vivo* have showed that POLDIP3-β overexpression significantly increased the proliferation and migration of HCC cells and promoted the growth of xenotransplantation. The above results have indicated that POLDIP3-β will be a potential target for the treatment of liver cancer.	Hepatocellular carcinoma (HCC)	Gene therapy based on adenoviral-mediated POLIDP3-βinhibition, CRISPR-Cas9 Gene Editing et al.

NM, no mention.

## Author contributions

JD designed the study. PH, LW, and NZ were major contributors to wrote the manuscript. JD and PH made substantial contributions to the design of the manuscript and revised it critically for important intellectual content. LW and PH created all the figures for the manuscript. NZ and HZ collected relevant data from TCGA and analyzed it. All authors have read and agreed to the published version of the manuscript.

## Footnotes

1
http://r2.amc.nl

## Abbreviations

ACSM1: Ac-CoA synthetase medium-chain family member 1
ADSS: adenylosuccinate synthase
AIG: anchorage independent growth
ALS: amyotrophic lateral sclerosis
AMP: adenosine monophosphate
ASD: autism spectrum disorder
ARNA: afferent renal nerve activity
ARDS: acute respiratory distress syndrome
SBC: secretory breast carcinoma
BBB: blood–brain barrier
CSAGA: complex sense-antisense architecture
DLAT: dihydrolipoamide S-acetyltransferase
ECM: extracellular matrix
ECT2: epithelial cell transformation sequence 2
EIF4G: eukaryotic initiation factor 4G
EJC: exon junction complex
EMT: epithelial mesenchymal transition
ERH: enhancer of the rudimentary homolog
FAK: focal adhesion kinase
IL-6: interleukin-6
IPP: intrapelvic pressure
ISG: interferon-stimulated genes
LIPT1: lipoyl-AMP is produced and lipoyltransferase 1
NOX4: NADPH oxidase 4
NSCLC: non-small cell lung cancer
MDM2: mouse double minute 2 homolog
MMP: matrix metalloproteinase
MYBL2: MYB proto-oncogene like 2
PCNA: proliferating cell nuclear antigen
PHD2: prolyl hydroxylase-2
POLDIP: polymeraseδ-interacting protein
RND: rho family GTPase
ROS: reactive oxygen species
RRM: RNA recognition motif
RSK1: ribosomal protein S6 kinase
RTEL1: telomere length 1
SP: substrate P
S6K1: ribosomal protein S6 kinase 1
TCA: tricarboxylic acid
TLS: DNA translesion synthesis
TNF-α: tumor necrosis factor-α
TREX: transcription-export
TRPV1: transient receptor potential vanilloid 1
VSMC: vascular smooth muscle cell.
